# Historical evolution of the scientific investigation of the placebo analgesic effect

**DOI:** 10.3389/fpain.2022.961304

**Published:** 2022-08-10

**Authors:** Fabrizio Benedetti

**Affiliations:** ^1^Neuroscience Department, University of Turin Medical School, Turin, Italy; ^2^Program in Hypoxia Medicine and Physiology, Plateau Rosà, Switzerland

**Keywords:** placebo, history of medicine, pharmacology, endogenous opioid analgesia, doctor—patient relationship

## Introduction

The placebo effect, particularlythe placeboanalgesic effect or placebo analgesia, is today a melting pot of concepts and ideas for neuroscience, and more in general for biological and medical sciences. Its study allows us to better understand several brain functions, ranging from anxiety to learning and from pain networks to higher cognitive functions. Likewise, it makes us understand how social interaction and the therapist-patient relationship are crucial in the therapeutic outcome, with particular emphasis on pain and analgesia. All pieces of information and all the advancement in knowledge we have gained so far stand on the shoulders of giants. Here I want to describe some fundamental steps over the past years that have allowed us to make intellectual progress in the understanding of the placebo phenomenon.

## Early observations

Henry Beecher was an anesthesiologist and pain therapist in Italy during World War II. On the battlefield he noted no correlation between the intensity of pain experienced by soldiers and the severity of the injuries (1). When Beecher returned to his practice in the United States after World War II, he compared the injuries of his patients with those of the soldiers, and found that the requirement for painkillers was substantially higher in civilian patients than in the soldiers on the battlefield (2), thus emphasizing the lack of relationship between the extension of the wounds and the perceived experience of pain. Beecher suggested that this discrepancy between soldiers and civilians could be attributable to the different meanings of the injuries. Whereas, the wound on the battlefield meant survival and returning home, the injured civilian was more concerned with diminishment of activities and loss of income. Beecher was one of the first clinician scientists who realized that the context around the patient makes the difference, and that the negative pain experience can turn into a positive experience. Thus, Beecher understood that the global pain experience is tightly linked to a variety of psychological factors associated to the injuries. For example, in soldiers, anxiety, emotional stress, grief from the loss of friends could be involved in different pain perceptions, as well as fear of losing the injured arm or being impotent because of injuries around the genitals.

It is within this context that Beecher's interest in the placebo effect emerged. In fact, Beecher often administered placebos to the soldiers, due to the lack of analgesics on the battlefield, along with verbal suggestions that they were powerful painkiller. Many of them responded to the placebo. In particular, he noted that an injection of saline solution had 90% of the effectiveness of morphine in relieving acute pain after injury, compared to civilian hospitals, where the placebo effect dropped to 70% of the effectiveness of morphine in pain after surgery (3, 4). Therefore, Beecher concluded that the placebo effect is related to the context in which it is being investigated. In his 1955 seminal paper, he reviewed 15 controlled trials involving 1,082 patients (5) and reported that placebos have an average significant effectiveness of 35.2 ± 2.2%. This average of one-third of patients responding to placebos has since permeated medical texts and teachings, although today this notion of one-third should be abandoned (6). In fact, the main criticism to Beecher's conclusion was that patients who received a placebo were not compared to those who received no treatment, thus making it impossible to rule out spontaneous remissions. Despite these limitations, Beecher's studies boosted the interest of the scientific community in the placebo effect.

In a study, Beecher compared different analgesics, like morphine, codeine, acetylsalicylic acid, and placebos (7), emphasizing the problem of the placebo effect and stressing the importance of placebo-controlled trials. He was particularly concerned with the problem of placebo reactors and non-reactors in clinical research in the design of clinical trials and their interpretation (3, 8). Not only did he consider placebo responsiveness of pain, but also of surgery. In another seminal paper (9), he emphasized how placebo effects can be powerful in both pharmacological and non-pharmacological treatments.

## Innovative ideas

Beecher's investigation of the placebo effect in the early 1950 s was further developed in the late 1950 s. Although the notion of a pharmacology of placebos was first approached by Wolf (10), the paper by Lasagna, Laties and Dohan (11) is one of the most important papers introducing the concept of a “pharmacology of placebo,” a really unusual and innovative idea at that time. Lasagna, Laties and Dohan (11) performed a straightforward comparison between drugs and placebos. This paper is important and fundamental, particularly by considering that placebo had always been conceived as a comparator in the setting of clinical trials or, alternatively, studied by psychologists as a model to understand the influence of mind over the body. By contrast, Lasagna et al. (11) approached the placebo from a mechanistic and pharmacological point of view by describing some of the characteristics of placebos compared to drugs. They performed a careful analysis of four main elements: peak effects, cumulative effects, carryover effects, and severity-related efficacy.

### Peak effects

One of the most important indices of pharmacologic activity is the time-effect relationship, namely, a maximal effect is achieved by a drug at a given point in time. In this paper (11), similar time-effect curves were found for aspirin and placebo in post-partum pain, although aspirin was much more effective than placebo.

### Cumulative effects

A feature of pharmacologic studies is the cumulative or build-up effect of repeated doses of a drug, which is likely to reflect increasing concentrations of drug in the body. In this article (11) it is shown that patients suffering from tuberculosis improved in pep and appetite after a placebo treatment that had been administered along with verbal suggestions of improved energy and appetite. It is worth noting that there was a cumulative effect over time, for both pep and appetite.

### Carryover effects

Not only can drugs cumulate in the body following repeated doses, but they can also produce long-lasting effects even after their administration is interrupted, the so-called carryover effects. Similar carryover effects were found for placebos in the same study on tuberculosis patients described above (11).

### Efficacy related to severity of disease

Another general characteristic of drugs is the inverse relationship of their effectiveness to the severity of a symptom such as pain. This holds true for placebos as well. Lasagna, Laties and Dohan (11) found that both aspirin and placebo showed lessened efficacy in patients with greater post-partum pain.

Besides the Lasagna et al.'s paper, the late 1950 s proved to be very productive and innovative years for placebo research. For example, some placebo surgery trials were performed for treatment of angina pectoris, a condition whereby there is inadequate blood supply of the heart (12, 13). In the 1950 s angina pectoris was treated frequently by ligation of the internal mammary arteries, as this procedure was believed to improve heart circulation through alternative routes of the blood into the heart. However, several years later no new blood vessel could be detected in the heart, thus making this kind of surgery questionable. Accordingly, Dimond et al. (12, 13) performed sham surgery, in which patients underwent the whole surgery for the ligation of the mammary arteries, but without actual ligation of the arteries. An improvement in pain outcomes, physical performance, and electrocardiogram was found in some patients. Overall, there was a substantial improvement in those who received real surgery as well as a substantial improvement in the placebo group.

## The dawn of placebo neurobiology

With the exception of a few studies, for example in animals (14), it was not before 1978 that a true neuroscience of the placebo effect emerged. Twenty years after Lasagna et al.'s paper (11), Levine et al. (15), gave the first mechanistic explanation of placebo analgesia, by showing that it could be antagonized by naloxone, an opioid antagonist, which strongly suggested the involvement of the endogenous opioid system. These findings have been criticized on many grounds, because of the lack of adequate control groups and the possibility that naloxone could be a hyperalgesic agent. Despite these limitations at that time, Levine et al. (15) were the first to give scientific credibility to the placebo phenomenon by unraveling the possible underlying biological mechanisms. In a sense, this study represented the passage from the psychological and clinical investigation of the placebo effect to its biological analysis. Indeed, the findings by Levine et al. (15) were confirmed by subsequent studies (6).

Along with this neuroscientific approach, in 1984, Fields and Levine (16) hypothesized that the placebo effect can be subdivided into opioid and non-opioid components. In particular, Fields and Levine suggested that different physical, psychological and environmental situations might affect the endogenous opioid systems differently. This concept was further supported by the finding that placebo analgesia does not always involve endogenous opioids (17), a notion that has been confirmed by more recent research (6). In the same years, Levine et al. (18, 19) showed that a hidden (unbeknownst to the patient) injection of a 6–8 mg morphine was comparable to an injection of saline solution in full view of the patient (placebo). In other words, telling the patient that a painkiller was being injected (with what was actually a saline solution) is as powerful as 6–8 mg of morphine. Thus, an open injection of morphine in full view of the patient is more effective than a hidden injection because in the latter the placebo component is absent, thereby emphasizing the crucial importance of the placebo effect in the therapeutic outcome.

## Conclusion

The rest of the story represents modern placebo research. It started in the 1990 s with a systematic scientific and biological investigation of the placebo effect. It relied on these early investigations and findings. It stands on the shoulders of giants. A myriad of studies have been performed so far, a variety of reviews have been written, and the main concept that has emerged over the past few years is that drugs and placebos share common mechanisms of action, an intriguing and challenging concept for neuroscience, pain research and, more in general, for the understanding of the human brain. Most of the modern findings and concepts, including some ethical issues, can be found in (6, 20).

## Author contributions

The author confirms being the sole contributor of this work and has approved it for publication.

## Conflict of interest

The author declares that the research was conducted in the absence of any commercial or financial relationships that could be construed as a potential conflict of interest.

## Publisher's note

All claims expressed in this article are solely those of the authors and do not necessarily represent those of their affiliated organizations, or those of the publisher, the editors and the reviewers. Any product that may be evaluated in this article, or claim that may be made by its manufacturer, is not guaranteed or endorsed by the publisher.
